# Advancing Breast Cancer Diagnosis: The Impact of Elastography Integration Into Breast Imaging Reporting and Data System (BIRADS) Categorization

**DOI:** 10.7759/cureus.65449

**Published:** 2024-07-26

**Authors:** George Asafu Adjaye Frimpong, Evans Aboagye, Emmanuel Asante, Osei Owusu-Afriyie, Ernest O Bonsu, Fairuuj Mahama

**Affiliations:** 1 Radiology, Kwame Nkrumah University of Science and Technology, Kumasi, GHA; 2 Radiology, Spectra Health Imaging and Interventional Radiology, Kumasi, GHA; 3 Research and Development, Spectra Health Imaging and Interventional Radiology, Kumasi, GHA; 4 Pathology, Kwame Nkrumah University of Science and Technology, Kumasi, GHA; 5 National Radiotherapy and Nuclear Medicine, Komfo Anokye Teaching Hospital, Kumasi, GHA

**Keywords:** treatment planning, stiffness, diagnostic accuracy, breast cancer, elastography

## Abstract

Objective: This study evaluates the impact of integrating elastography into the Breast Imaging Reporting and Data System (BIRADS) categorization on breast cancer diagnostics in an African population. It explores the association and agreement between traditional BIRADS and those modified by elastography, as well as between quantitative and qualitative elastography methods.

Methods: A total of 200 participants who underwent breast imaging as part of their diagnostic evaluation for breast lesions were included in the study. Participant characteristics, including age distribution and indicators for breast cancer diagnoses, were analyzed. Brightness mode (B-mode) findings without elastography were assessed using the BIRADS classification. Elastography was integrated into the BIRADS categorization to evaluate its impact on breast cancer diagnostics. The association and agreement between BIRADS with and without elastography were analyzed.

Results: Participants predominantly aged 40-49 showed significant staging differences with the integration of elastography. Traditional B-mode staging identified 29 (49%) of participants in BIRADS stage IV and 14 (23%) in stage V, whereas elastography adjusted these figures significantly, enhancing diagnostic refinement. There was a fair agreement between BIRADS with and without elastography (kappa = 0.322), while a substantial agreement was found between quantitative and qualitative elastography (kappa = 0.674).

Conclusion: The results of the study provide evidence that the integration of elastography into BIRADS categorization can significantly improve the accuracy of breast cancer diagnosis in African women. Elastography enhanced lesion characterization, supporting more personalized and precise clinical management. Continued research is needed to fully integrate elastography into routine diagnostic workflows and understand its broader clinical implications in Africa.

## Introduction

Breast cancer remains one of the most significant health challenges worldwide. In 2022, 2.3 million women were diagnosed with breast cancer, with 670,000 breast cancer-related deaths that same year [1]. Despite a lower incidence of breast cancer in Sub-Saharan Africa, mortality rates in this region are disproportionately high compared to developed countries [2,3]. Early detection and accurate characterization of breast lesions are crucial for effective treatment and improved survival rates [4].

Traditional diagnostic methods, primarily relying on mammography and ultrasound, are standardized through the Breast Imaging Reporting and Data System (BIRADS) categorization [5]. However, these conventional techniques often face limitations, especially in cases of dense breast tissue, which is prevalent among a substantial portion of the female population [6]. This limitation underscores the necessity for advancements in diagnostic methodologies that can provide more precise and reliable breast lesion characterization.

The introduction of elastography into the breast cancer diagnostic pathway represents a significant advancement. Elastography is an imaging modality that assesses tissue stiffness, a parameter often associated with malignancy [7]. Malignant lesions tend to be stiffer than benign lesions due to tumors' dense cellular and stromal composition. By measuring tissue stiffness, elastography offers a complementary diagnostic criterion, potentially enhancing the sensitivity and specificity of breast cancer diagnostics beyond what is achievable with traditional imaging alone [8].

Integrating elastography with BIRADS categorization promises a more nuanced approach to diagnosing breast lesions [9,10]. Despite its potential, the impact of this integration on diagnostic outcomes has not been fully explored. This knowledge gap is particularly pronounced outside of high-resource healthcare settings, where access to advanced imaging technologies like elastography may be limited. Therefore, investigating the effect of elastography’s integration into BIRADS categorization is not only of scientific interest but also of practical importance in improving breast cancer care.

This study evaluates the diagnostic shift from the initial experience with the integration of shear wave elastography (SWE) into BIRADS categorization. Specifically, it seeks to determine whether elastography can refine the accuracy of lesion characterization, leading to more precise categorizations and, consequently, more informed clinical decisions in resource-limited settings.

## Materials and methods

This retrospective study involved a review of the medical records of 200 consecutive patients who underwent elastography as part of their diagnostic evaluation for breast lesions at Spectra Health and Imaging and Interventional Radiology, in Kumasi, Ghana. Data extracted included age, imaging findings, and the BIRADS category (with and without elastography). Adult women aged ≥18 years and <30 years who received an ultrasound examination and adult women aged ≥30 years who received both a mammogram and an ultrasound between June 2020 and December 2023 were included in the study. In addition, 59 of 200 cases with available histopathological findings after biopsy were included in a concordance analysis. The study excluded women with breast implants, those with insufficient medical records, and those lacking imaging data required for analysis.

Examination with SWE and brightness-mode (B-mode) ultrasound

**Figure 1:**
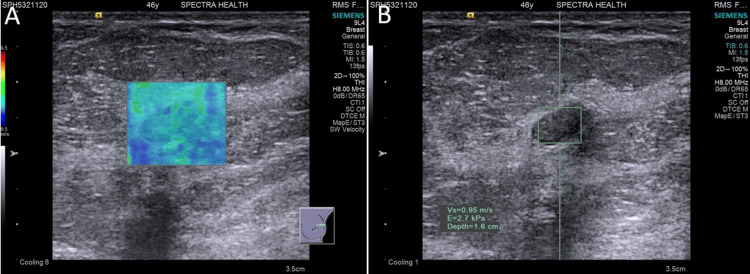
Benign lesion: (A) Qualitative elastography shows low stiffness with a predominantly blue color map. (B) Quantitative elastography shows low stiffness with an acoustic radiation force impulse (ARFI) velocity of 0.95 m/s.

**Figure 2:**
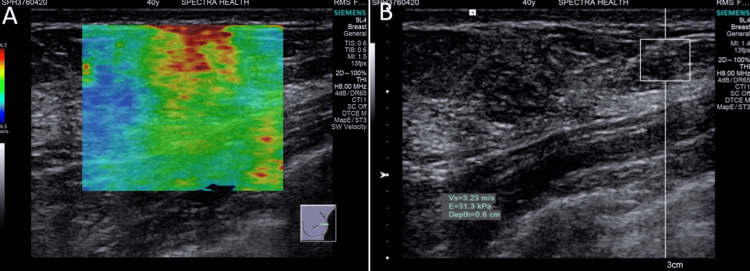
Suspicious lesion: (A) Qualitative elastography shows moderate stiffness with an area of red on the color map. (B) Quantitative elastography shows moderate stiffness with an acoustic radiation force impulse (ARFI) velocity of 3.34 m/s.

**Figure 3:**
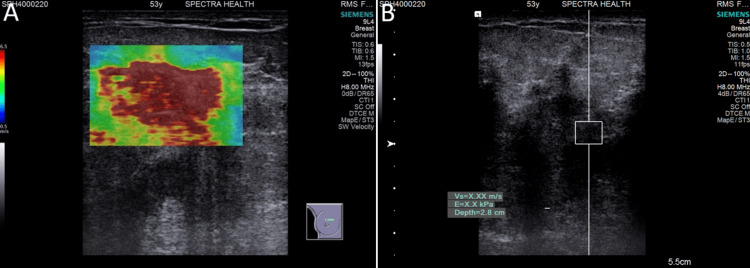
Malignant lesion: (A) Qualitative elastography shows very high stiffness with a predominantly red color map. (B) Quantitative elastography shows very high stiffness with acoustic radiation force impulse (ARFI) velocity beyond the measurable range recorded as X.XX m/s (≥ 6 m/s). X.XX m/s denotes SWE exceeding the default upper limit of ultrasound scanner (≥6 m/s).

The patients were examined while lying in the supine position with their breasts exposed and both hands placed behind the head to expose the axilla. The Siemens Acuson S3000 Ultrasound System (Siemens Medical Solutions USA, Inc., PA, USA) was used for each procedure. The breast lesions were assessed using a high-frequency linear transducer (7-12 MHz) in both radial and anti-radial planes. The assessment included qualitative and quantitative elastography findings and B-mode findings for each lesion. SWE was performed to obtain virtual touch imaging (VTI^TM^) and virtual touch quantification (VTQ^TM^) values (Figures 1-3). Two radiologists with more than 15 years of experience in sonography and five years of experience using acoustic radiation force impulse (ARFI) interpreted all of the studies.

BIRADS categorization

Traditional BIRADS categorization was based on findings from mammography and ultrasound according to the established American College of Radiology guidelines. Categories ranged from 0 (incomplete) to VI (known biopsy-proven malignancy), with each category indicating a different level of suspicion for malignancy.

Statistical analysis

For the association between elastography findings and BIRADS categorization shifts, Chi-square tests were performed. Agreement between BIRADS categorizations with and without elastography and qualitative and quantitative elastography methods were also performed with kappa statistics. P-values < 0.05 were considered statistically significant. All statistical analyses in this study were performed using the GraphPad Prism software version 8.0 for Windows (GraphPad Software, San Diego, CA, USA).

## Results

Age distribution and indicators of breast cancer diagnoses among the study participants

**Figure 4:**
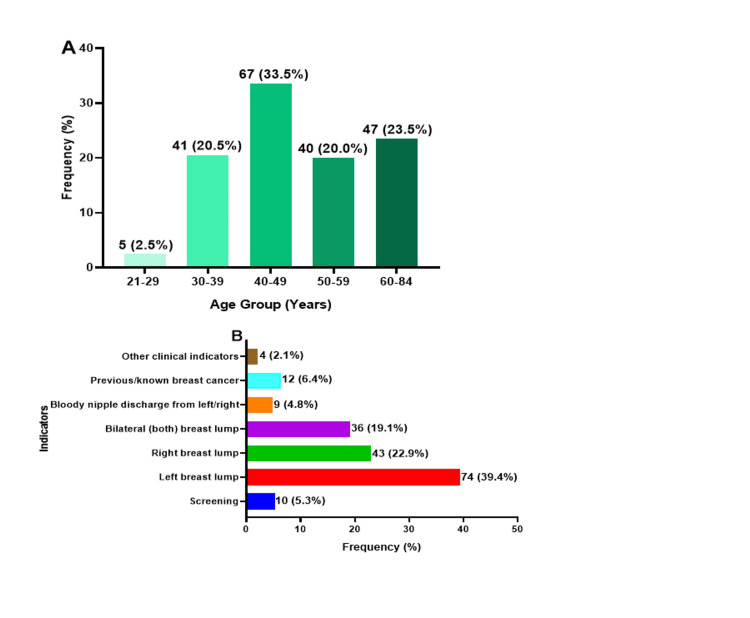
(A) Age distribution among the study participants and (B) indicators of breast cancer diagnosis.

The study included 200 participants, with 67 (33.5%) between 40 and 49 years old, followed by 47 (23.5%) in the 60-84 age range. Among 188 patients with known indicators of breast cancer diagnoses, 74 (39.4%) had lumps in the left breast, while 43 (22.9%) had lumps in the right breast. Nine (4.8%) patients reported bloody discharge from their breasts (Figure 4).

B-mode findings among the study participants

**Figure 5:**
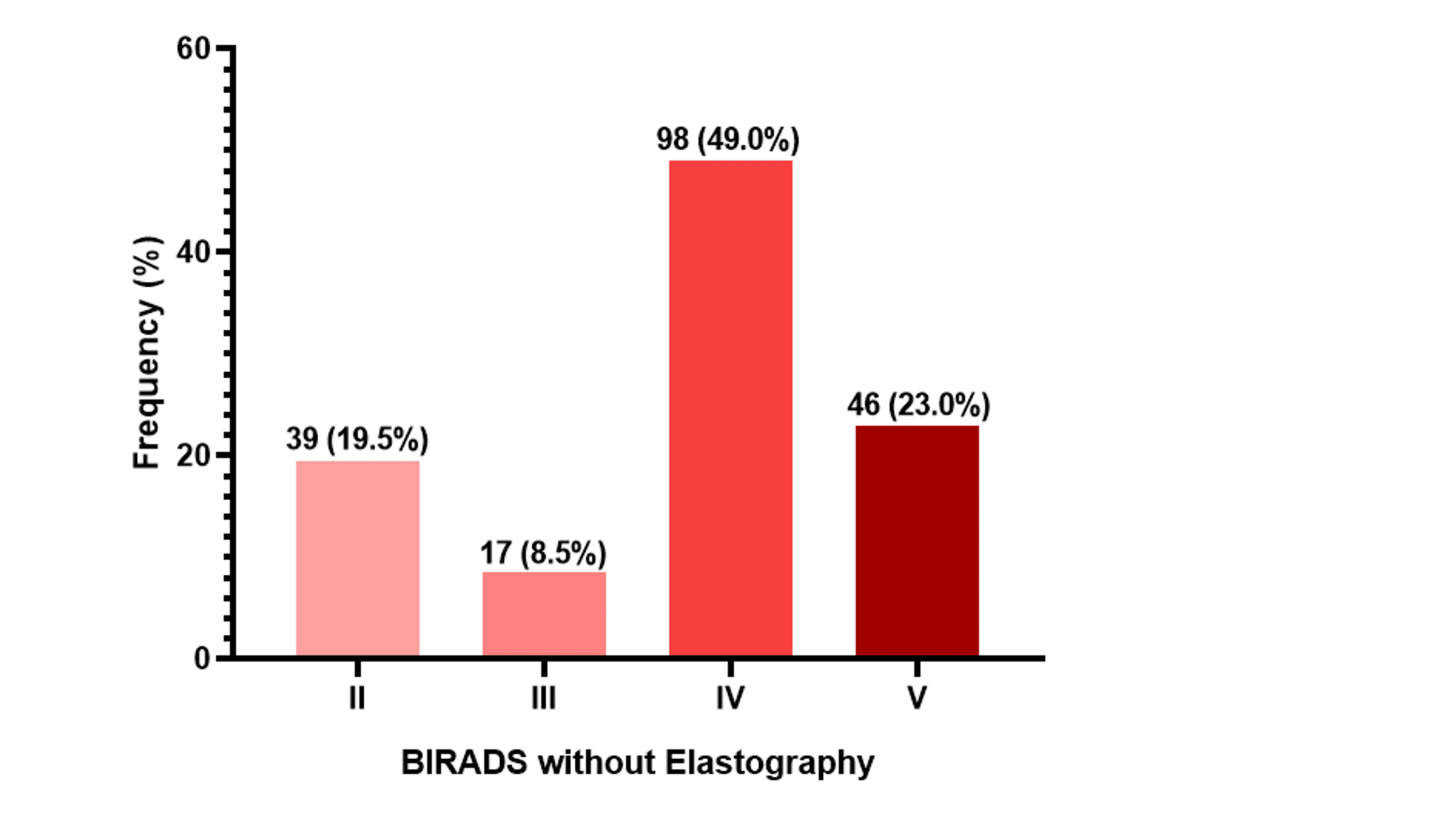
B-mode findings.

The B-mode findings indicated that almost half of the participants, 98 (49.0%), were classified as stage IV according to the BIRADS classification. This was followed by stage V, comprising 46 (23.0%) participants. In addition, 39 (19.5%) were categorized as stage II, while 17 (8.5%) participants were categorized as stage III (Figure 5).

Association between quantitative and qualitative elastography

**Figure 6:**
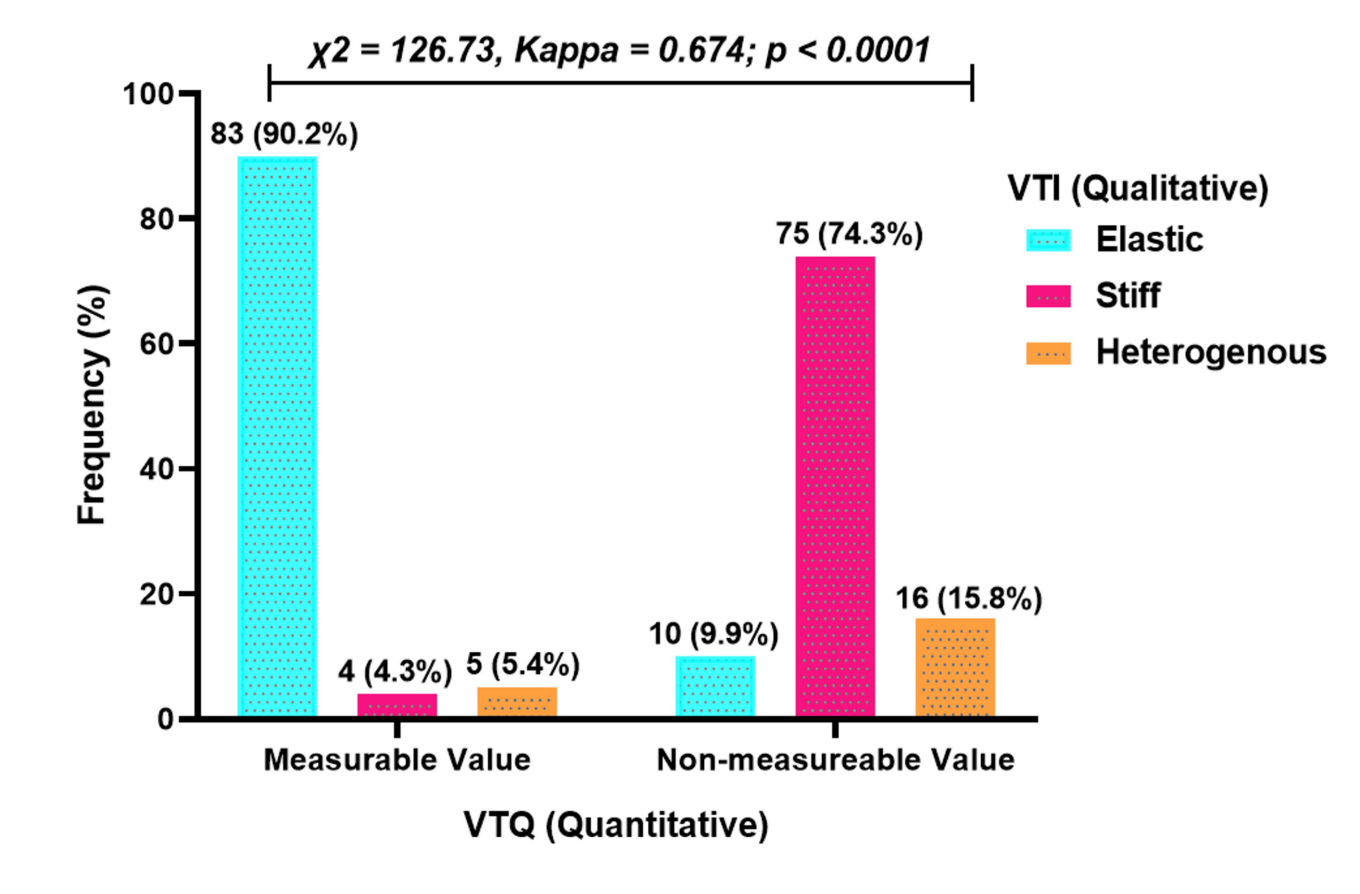
Association between quantitative and qualitative elastography A p-value less than 0.05 was considered statistically significant.

In terms of quantitative and qualitative elastography findings, 83 (90.2%) cases were classified as elastic, four (4.3%) as stiff, and five (5.4%) as heterogeneous. Among those with non-measurable values (≥6.5 m/s), 75 (74.3%) were classified as stiff, 10 (9.9%) as elastic, and 16 (15.8%) as heterogeneous. A significant association was observed between quantitative and qualitative elastography methods (p < 0.0001), with a moderate agreement (kappa = 0.674) (Figure 6).

Association between BIRADS with and without elastography (all cases)

Among the 39 participants initially diagnosed as stage II without elastography, 32 (82.1%) remained in stage II after elastography, while 3 (7.7%) were upgraded to stage III and 4 (10.3%) to stage IV. For participants initially diagnosed as having BIRADS III without elastography, six (35.3%) remained in stage III, five (29.4%) were downgraded to stage II, and six (35.3%) were upgraded to stage IV after elastography.

**Figure 7:**
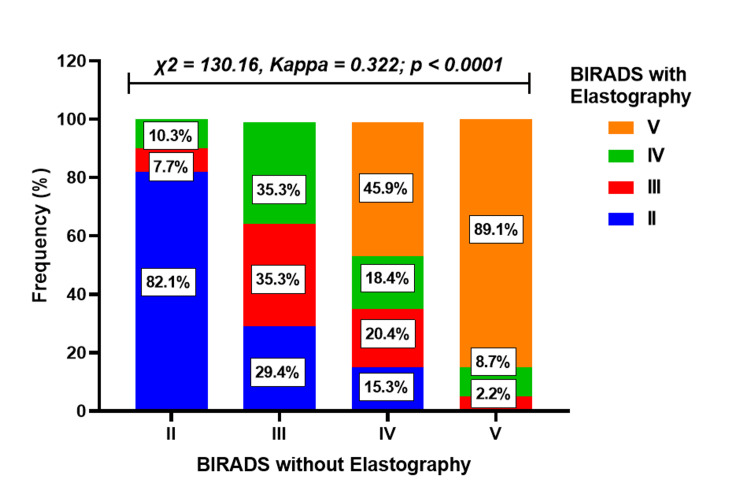
Association and agreement between BIRADS without elastography and BIRADS with elastography diagnostic methods. A p-value less than 0.05 was considered statistically significant. BIRADS: Breast Imaging Reporting and Data System

For patients initially diagnosed as stage IV without elastography, 18 (18.4%) remained in stage IV, while 15 (15.3%) were downgraded to stage II, 20 (20.4%) to stage III, and 45 (45.9%) were upgraded to stage V. Among participants initially diagnosed as stage V without elastography, 41 (89.1%) remained in stage V, one (2.2%) was downgraded to stage III, and four (8.7%) to stage IV with elastography. The differences in staging between BIRADS with and without elastography were statistically significant (p < 0.001), and the agreement between the two methods was fair, with a kappa value of 0.322 (Figure 7).

Association between BIRADS with and without elastography for histologically confirmed cancers

**Figure 8:**
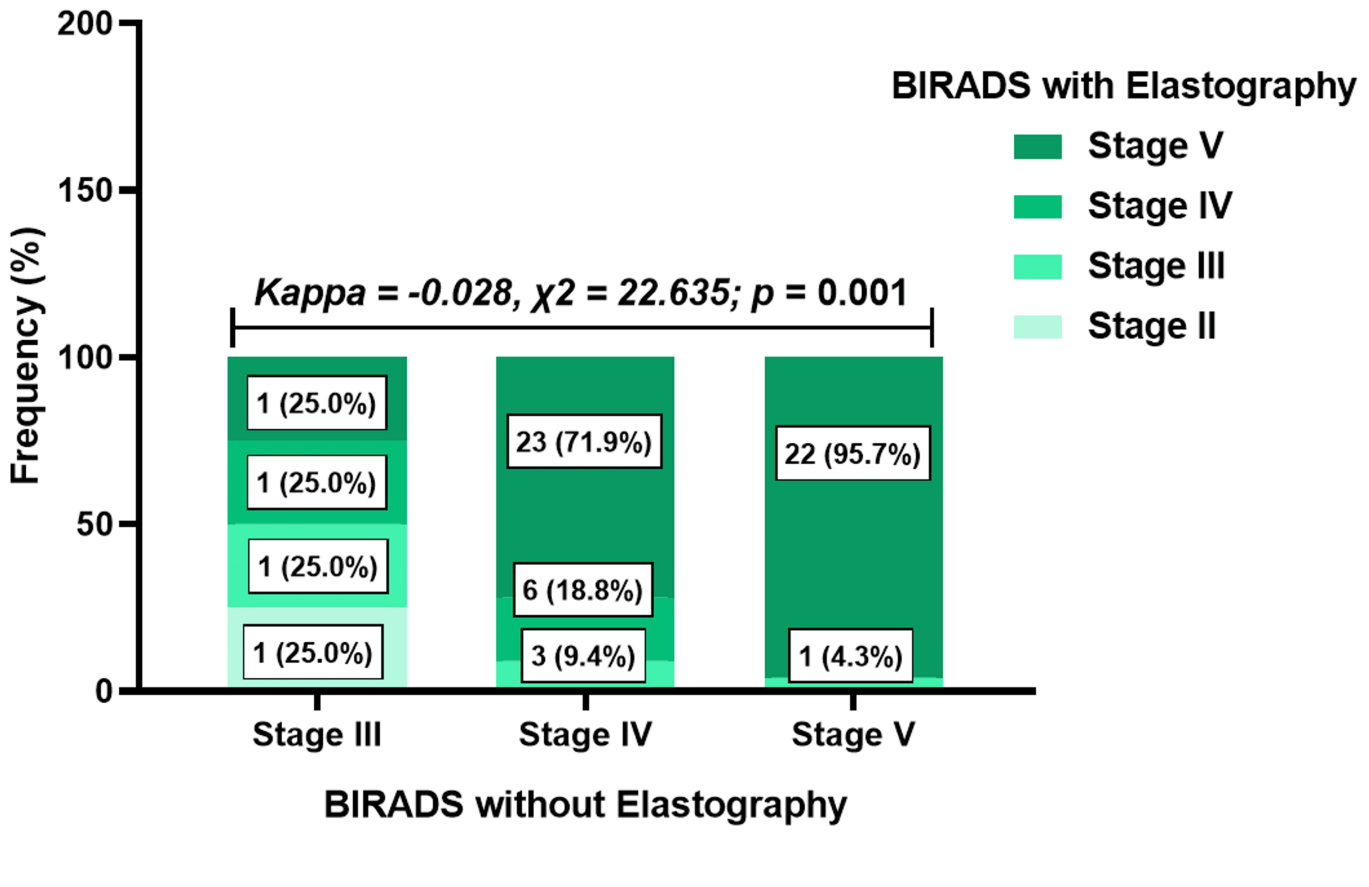
Impact of elastography on ultrasound BIRADS categorization for the histologically confirmed cancers BIRADS: Breast Imaging Reporting and Data System

Out of the 200 cases, 59 (29.5%) biopsy-confirmed cancers were available at the time of the study. When comparing findings from BIRADS with and without elastography in these biopsy-confirmed cases, it was observed that two out of four cases initially classified as BIRADS III without elastography were upgraded to BIRADS IV, and V after elastography, while one (25.0%) case was downgraded to BIRADS II. Among 32 BIRADS IV cases without elastography, elastography upgraded 23 (71.9%) to BIRADS V and downgraded three (9.4%) to BIRADS III. In addition, of 23 cases initially classified as BIRADS V without elastography, only one (4.3%) was downgraded to BIRADS III, with the remaining cases maintaining their status as BIRADS V cases. These results indicated a significant difference between BIRADS with and without elastography for the histologically confirmed cancers (p = 0.001). The kappa statistics indicated poor agreement between BIRADS without elastography and BIRADS with elastography (kappa = -0.028) (Figure 8).

**Figure 9:**
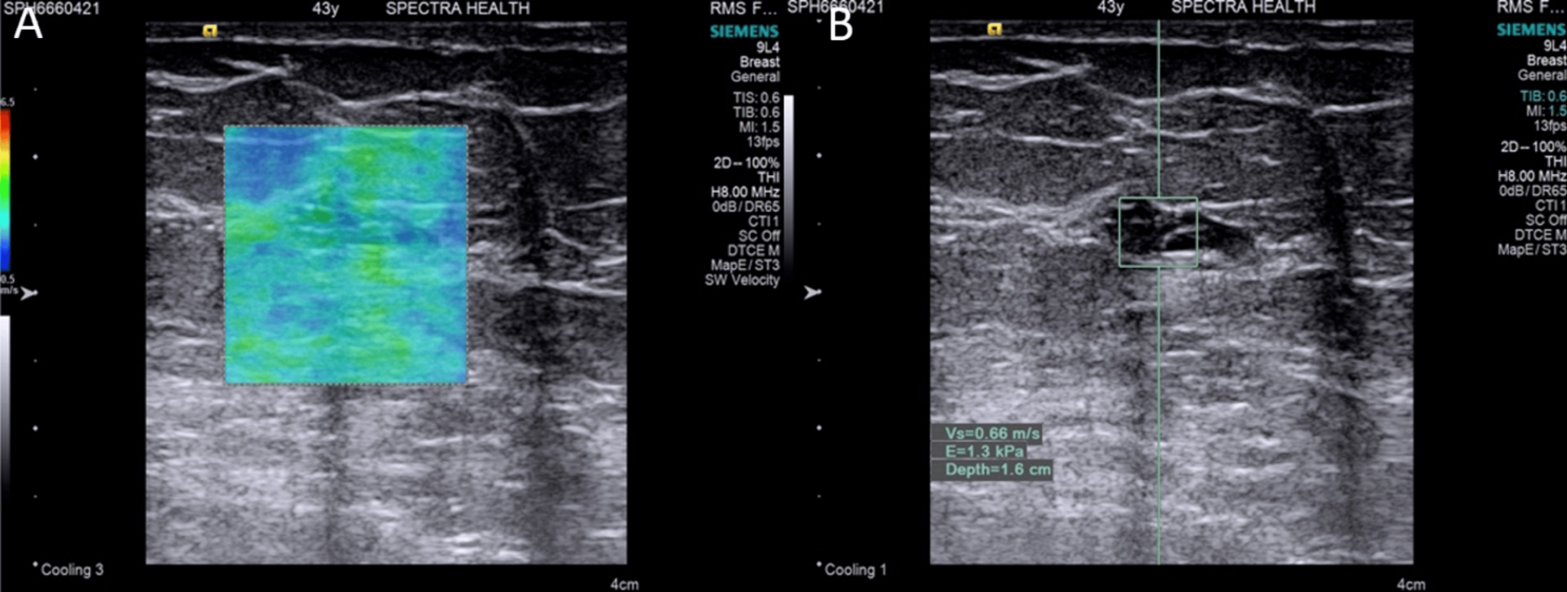
Histology confirmed carcinoma (invasive carcinoma): (A) Qualitative elastography shows low stiffness with a predominantly green color map. (B) Quantitative elastography shows low stiffness with an acoustic radiation force impulse (ARFI) velocity of 0.66 m/s.

In five histologically confirmed cases, BIRADS categorization was downgraded with elastography. Notably, three of these cases involved complex breast cysts - two type 2 and one type 4. The remaining tumors had areas of cystic necrosis. This observation highlighted the inherent characteristics of cystic malignancies, which typically exhibited less stiffness compared to solid tumors (Figure 9).

## Discussion

The integration of elastography into BIRADS categorization was evaluated in this study to assess its impact on the diagnostic process, staging accuracy, and treatment planning of breast lesions. The results provide valuable insights into the potential benefits of incorporating elastography in the assessment of breast cancer. The study participants' age distribution revealed a higher representation of women in the 40-49 age range, which aligns with the known trend of increased breast cancer incidence in this age group [11,12]. The most common indicator for breast cancer diagnoses was the presence of breast lumps, particularly in the left breast, which is consistent with previous studies [13,14].

The B-mode ultrasound findings without elastography revealed that almost half of the participants were classified as having BIRADS IV, followed by BIRADS V lesions. According to the National Comprehensive Cancer Network, BIRADS IV lesions are characterized as suspicious for malignancy, presenting a wide range of malignancy probabilities ranging from 2% to 95% [15]. These findings stress the need for increased awareness and utilization of screening programs to detect breast cancer at earlier stages to improve treatment outcomes, quality of life, and survival rates of patients [16,17].

Elastography’s utility in differentiating between benign and malignant lesions, as well as its potential to augment traditional imaging techniques to avoid unnecessary breast biopsies, have been studied [18,19]. For example, in the study conducted by Ng et al., the utilization of elastography with a cutoff of ≥80 kPa resulted in the downgrading of 95.5% of the BIRADS IVa lesions to BIRADS III, consequently obviating the necessity for a biopsy [20]. In this study, all BIRADS categories with and without elastography were considered to reflect elastography's potential in refining breast lesion assessment. In this present study, SWE downgraded about a quarter of BIRADS IV lesions, preventing patients from undergoing biopsy. For concordance analysis of biopsy-confirmed cases, the majority of BIRADS V lesions without elastography were maintained after integrating SWE.

While malignancies typically present with increased stiffness, cystic malignancies may not conform to this pattern due to their unique structural composition [21,22]. Biopsy-confirmed cystic malignancies were reported to have downgraded BIRADS stages after integrating SWE. This finding confirms that elastography might be limited in accurately categorizing cystic malignancies, as observed in breast and parotid gland applications [23,24]. Therefore, radiologists must employ a combination of imaging modalities and clinical judgment when evaluating such cases.

Quantitative and qualitative elastography demonstrated consistent associations with stiffness in breast cancer lesions. Most measurable values (<6 m/s) were classified as soft, while non-measurable values (≥6 m/s) were predominantly stiff. These findings align with the expected characteristics of breast cancer, where malignant lesions are typically stiffer than surrounding healthy tissue [25]. The agreement between quantitative and qualitative SWE further supports their reliability in evaluating stiffness and demonstrates elastography's potential to distinguish between benign and malignant lesions, leading to more informed clinical decisions and earlier intervention strategies [26]. Elastography's role in reducing diagnostic uncertainty, particularly in cases involving dense breast tissue, underscores its value as a complementary tool to conventional imaging techniques [27].

This study is not without limitations. The sample size was relatively small, which may restrict the statistical power and generalizability of the findings. In addition, only a few cases with confirmed biopsies were available due to patients choosing not to undergo biopsy, financial constraints, and biopsies being taken at different institutions. The study did not evaluate the impact of elastography on treatment decisions and patient outcomes, and further research is necessary in this area.

## Conclusions

The current study demonstrates a significant diagnostic shift with the inclusion of elastography, increasing the growing body of evidence supporting elastography’s role in enhancing breast lesion characterization. It also pioneers its use in an African population. The findings highlight the importance of incorporating elastography into clinical practice, including improved diagnostic accuracy, enhanced lesion characterization, and potential for early detection. The study also reveals a critical observation in the application of elastography for cystic malignancies, where BIRADS was occasionally downgraded despite histological confirmation of cancer. This observation suggests that while elastography is a valuable addition to breast cancer diagnostics, it must be used judiciously, particularly in cases of complex cystic lesions. Further research is necessary to refine the use of elastography, ensuring it complements other diagnostic methods effectively.
